# Validation of the Edinburgh Postpartum Depression Scale in a Population of Adult Pregnant Women in Mexico

**DOI:** 10.14740/jocmr1883w

**Published:** 2014-07-28

**Authors:** Cosme Alvarado-Esquivel, Antonio Sifuentes-Alvarez, Carlos Salas-Martinez

**Affiliations:** a Biomedical Research Laboratory, Faculty of Medicine and Nutrition, Juarez University of Durango State, Durango, Mexico; b General Hospital, Secretary of Health, Durango, Mexico

**Keywords:** Pregnancy, Depression, Validation study, Mexico

## Abstract

**Background:**

The Edinburgh postnatal depression scale (EPDS) is useful for screening depression in puerperal women as well as women during pregnancy. However, such instrument should be validated in a given language before it can be used. There is not validated Mexican version of the EPDS for use in adult pregnant women. Therefore, we sought to validate a Spanish translated Mexican version of the EPDS in a population of adult pregnant women.

**Methods:**

One hundred fifty-eight adult women (mean age: 28 ± 6.8 years; range: 18 - 45 years) within their 2 - 9 months of pregnancy attending routine prenatal consultations in a public hospital in Durango City, Mexico were studied. All pregnant women submitted a Spanish translated Mexican version of the EPDS. In addition, participants were assessed for major and minor depression by using the DSM-IV criteria.

**Results:**

Of the 158 pregnant women studied, 11 had major depression and 26 had minor depression by the DSM-IV criteria. The best EPDS score for screening combined major and minor depression in adult pregnant women was 9/10. This threshold showed a sensitivity of 75.7%, a specificity of 74.4%, a positive predictive value of 50.8%, a negative predictive value of 94.7% and an area under the curve of 0.89 (95% confidence interval: 0.71 - 1.06).

**Conclusion:**

The Mexican version of the EPDS can be considered for screening depression in Mexican adult pregnant women whenever a cut-off score of 9/10 is used.

## Introduction

Depression in women may occur during pregnancy and its frequency and severity vary among women populations around the world [1-3]. In a recent review, researchers found that depression during pregnancy is highly prevalent, is associated with negative outcome in the newborn and remained scanty studied [4]. In a meta-analysis of 36 studies, researchers found that elevated depression levels during early- to mid-pregnancy increased the risk of preterm birth and small-for-gestational-age [5]. However, the rate of detection of depression during pregnancy is generally low and many depressed women are not diagnosed and lack suitable support [6, 7]. Depression during pregnancy has epidemiological importance because the prevalence of depression during pregnancy can be higher than the one in the postnatal period [8, 9]. In fact, depression during pregnancy was a factor associated with postnatal depression in a study in our region [10]. The Edinburgh postnatal depression scale (EPDS) is an instrument used for screening depression during their postnatal period [11-13] or during pregnancy [8, 9, 14, 15]. However, translations of the EPDS to languages other than the original English version [11] should be validated. Studies on validation of the EPDS are important to determine the optimal cut-off scores of the instrument for screening depression in a given population. There is not a validated Mexican version of the EPDS for the use in pregnant women. Therefore, the present study was aimed to validate a Spanish translated Mexican version of the EPDS (Supplementary 1, www.jocmr.org) in a population of adult pregnant women in Durango, Mexico.

## Methods

### Selection and description of participants

Adult pregnant women attending routine prenatal consultations in a public hospital (General Hospital of the Secretary of Health) in Durango City, Mexico were studied from March to December 2013. Inclusion criteria for enrollment in the study were pregnant women within their 1 - 9 months of pregnancy, aged 18 years and older, of any occupation, socioeconomic status, and who accepted to participate. Selection of participants was performed at random. In total, 158 women were included in the study. The studied women had a mean age of 28 ± 6.8 years old (range: 18 - 45 years), and had a low socioeconomic status. They were evaluated once within their 2 - 9 months (median: 8 months) of pregnancy. Of the 158 women studied, 26 were in their first pregnancy and 132 were in their second to eighth pregnancy.

### Evaluation of the EPDS in pregnant women

The EPDS used in pregnant women was constructed from the original English version [11] and a Mexican version [13] of the instrument. Special care was taken to use words currently spoken for the general population in Mexico. In addition, we revised that the meaning of the words and the general structure of the Mexican version of EPDS were in close agreement with those of the original English version. The revised version of the EPDS had only one change from the previous Mexican version [10, 13]. In question number 8, the word “desgraciada” was replaced with “miserable”. Such a change was performed to make a more accurate translation from the original version of the instrument [11] and to improve the meaning of the question in the Spanish language currently spoken in Mexico. All participants completed the revised self-administered Mexican version of the EPDS. Participants were also interviewed by a psychiatrist to examine major and minor depression by using the DSM-IV criteria [16]. Both examinations (EPDS and psychiatric interview) were performed during the same day to every participant. The psychiatrist who assessed depression was blind to the EPDS scores. Neither the psychiatrist nor the gynecologist who applied the EPDS performed the data analysis.

### Statistical analysis

The statistical analysis was performed with the aid of the SPSS version 15.0 software. Sensitivity, specificity, and positive and negative predictive values of the evaluated EPDS were obtained. The best cut-off scores of the revised Mexican version of the EPDS for screening depression in adult pregnant women were obtained by drawing a receiver operating characteristic curve.

### Ethical aspects

This study was approved by the Ethical Committee of the General Hospital of the Secretary of Health in Durango City, Mexico. The purpose and procedures of the study were explained to all pregnant women, and a written informed consent was obtained from all of them.

## Results

**Figure 1:**
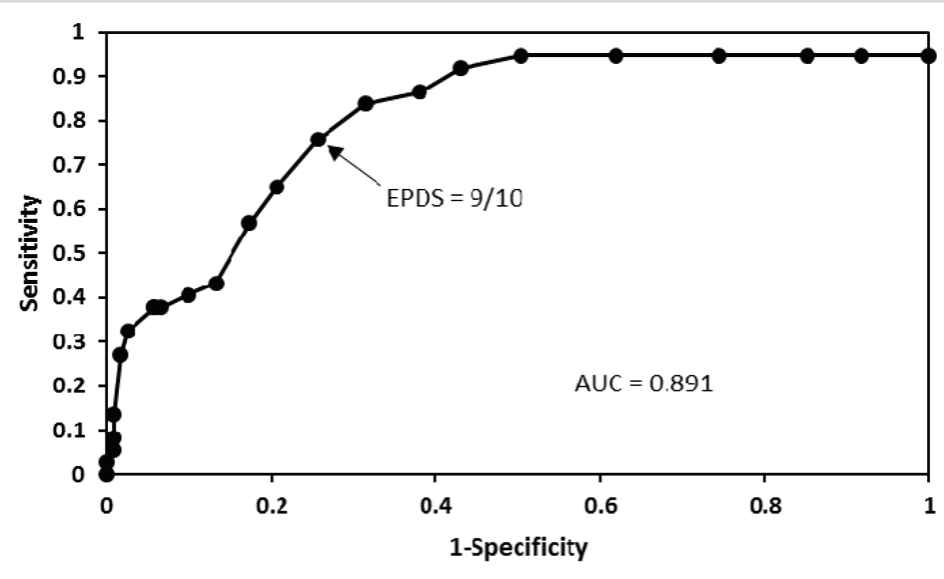
A receiver operating characteristic curve that shows different cut-off points of the EPDS in pregnant women. Good performance of the Mexican version of the EPDS in these women was found at 9/10 cut-off point.

Of the 158 pregnant women studied, 11 had major depression and 26 had minor depression according to the DSM-IV criteria. Results of sensitivity and specificity for different EPDS scores found in the 158 women are shown in Table 1. As seen in Figure 1, the receiver operating characteristic curve showed that the best sensitivity and specificity of the Mexican version of the EPDS in pregnant women was found at 9/10 score. At this threshold, we found a sensitivity of 75.7% and a specificity of 74.4%. The area under the curve was 0.89 (95% confidence interval: 0.71 - 1.06). Increasing the threshold to 10/11, the sensitivity was reduced to 64.9%, but the specificity increased to 79.3%. While lowering the threshold to 8/9, the sensitivity increased to 83.8%, but the specificity was reduced to 68.6%. Of the 37 women with depression by the DSM-IV criteria, 32 were positive and five negative in the EPDS. While of the 121 women without depression by the DSM-IV criteria, 90 were negative and 31 positive in the EPDS. Thus a positive predictive value of 50.8% and a negative predictive value of 94.7% for the EPDS were obtained. Depressed women were treated either with sertraline or psychotherapy.

**Table 1:** Sensitivity and Specificity of the Mexican Version of EPDS at Different Thresholds As Compared With DSM-IV Results in Adult Pregnant Women

EPDS score	Sensitivity (%)	Specificity (%)
0 - 1	94.6	0
1 - 2	94.6	8.3
2 - 3	94.6	14.9
3 - 4	94.6	25.6
4 - 5	94.6	38
5 - 6	94.6	49.6
6 - 7	91.9	57
7 - 8	86.5	62
8 - 9	83.8	68.6
9 - 10	75.7	74.4
10 - 11	64.9	79.3
11 - 12	56.8	82.6
12 - 13	43.2	86.8
13 - 14	40.5	90.1
14 - 15	37.8	93.4
15 - 16	37.8	94.2
16 - 17	37.8	94.2
17 - 18	32.4	97.5
18 - 19	27	98.3
19 - 20	13.5	99.2
20 - 21	8.1	99.2
21 - 22	5.4	99.2
22 - 23	2.7	100
24 - 25	0	100

## Discussion

There is a lack of a validated instrument to screen depression in pregnant women in Mexico. In the present study, we sought to validate the EPDS in a population of pregnant women in Northern Mexico. Validation of the EPDS in pregnant women in our population is an important first step before the instrument can be used for screening depression in our region. The use of a validated EPDS may allow an easier identification of depressed pregnant women who should be examined by a psychiatrist. We found that the Mexican version of the EPDS can be successfully used to screen depression in a Mexican population of pregnant women. This instrument performed good with a threshold of 9/10. Such score is lower than the 12/13 score described in the original English version of the EPDS for postnatal depression [11]. Although the optimal cut-off score of 9/10 obtained in the present study is lower than that of 12/13 found in the original version, there is a comparable sensitivity and specificity among both studies. The optimal threshold of 12/13 reported in the original version of the EPDS has a sensitivity of 86% and a specificity of 78% [11] and our 9/10 score has a sensitivity of 75.7% and a specificity of 74.4%. To the best of our knowledge, there is not a validation study of the EPDS in Spanish language in pregnant women. Therefore, we cannot compare our results with others in the Spanish language context. Comparison of the threshold found in the present study with those reported in other validation studies in pregnant women using EPDS translated in languages other than Spanish in several countries show substantial differences. For instance, a validation study of the EPDS in women with high risk pregnancies in France showed an optimal cut-off score of 11.5 [17] which is slightly higher than the 9/10 score obtained in our study. The optimal cut-off score found in the present study is also slightly lower than the optimal 11 or higher score for detecting major depressive disorders found in pregnant women in Lithuania [18]. Similarly, the optimal 9/10 score obtained with the EPDS in pregnant women in Mexico is lower than the optimal EPDS 12/13 score found in Taiwanese pregnant women [19] and the optimal EPDS 13 or higher scores found in Maltese pregnant women [20] and Swedish pregnant women [21]. In contrast, our 9/10 score is similar to the 9.5 score found in Chinese pregnant women [22], the 10 score for detection of major depressive disorders in American pregnant women [23], and the 10 score for detection of major depressive episode in women during the second and third trimesters of pregnancy in The Netherlands [14]. Variability of optimal EPDS scores among studies stresses the need of performing validation studies of such instrument before it can be used for screening depression in pregnant women in a given country. The use of a validated EPDS is important since it contributes to getting reliable results in screenings for depression. In addition, a reliable detection of depressed pregnant women by screenings may aid for an optimal planning of diagnostic and treatment measures for depression by psychiatrists and those involved in making public health policies.

### Conclusions

The Mexican version of the EPDS performs well for screening depression in Mexican adult pregnant women. Recommended EPDS cut-off score in Mexican adult pregnant women within their 2 - 9 months of pregnancy is 9/10.
